# Brain Rhythms Reveal a Hierarchical Network Organization

**DOI:** 10.1371/journal.pcbi.1002207

**Published:** 2011-10-13

**Authors:** G. Karl Steinke, Roberto F. Galán

**Affiliations:** 1 Department of Biomedical Engineering, School of Engineering, Case Western Reserve University, Cleveland, Ohio, United States of America; 2 Department of Neurosciences, School of Medicine, Case Western Reserve University, Cleveland, Ohio, United States of America; Indiana University, United States of America

## Abstract

Recordings of ongoing neural activity with EEG and MEG exhibit oscillations of specific frequencies over a non-oscillatory background. The oscillations appear in the power spectrum as a collection of frequency bands that are evenly spaced on a logarithmic scale, thereby preventing mutual entrainment and cross-talk. Over the last few years, experimental, computational and theoretical studies have made substantial progress on our understanding of the biophysical mechanisms underlying the generation of network oscillations and their interactions, with emphasis on the role of neuronal synchronization. In this paper we ask a very different question. Rather than investigating *how* brain rhythms emerge, or whether they are necessary for neural function, we focus on *what* they tell us about *functional* brain connectivity. We hypothesized that if we were able to construct abstract networks, or “virtual brains”, whose dynamics were similar to EEG/MEG recordings, those networks would share structural features among themselves, and also with real brains. Applying mathematical techniques for inverse problems, we have reverse-engineered network architectures that generate characteristic dynamics of actual brains, including spindles and sharp waves, which appear in the power spectrum as frequency bands superimposed on a non-oscillatory background dominated by low frequencies. We show that all reconstructed networks display similar topological features (e.g. structural motifs) and dynamics. We have also reverse-engineered putative diseased brains (epileptic and schizophrenic), in which the oscillatory activity is altered in different ways, as reported in clinical studies. These reconstructed networks show consistent alterations of functional connectivity and dynamics. In particular, we show that the complexity of the network, quantified as proposed by Tononi, Sporns and Edelman, is a good indicator of brain fitness, since virtual brains modeling diseased states display lower complexity than virtual brains modeling normal neural function. We finally discuss the implications of our results for the neurobiology of health and disease.

## Author Summary

The fact that the brain generates weak but measurable electromagnetic waves has intrigued neuroscientists for over a century. Even more remarkable is the fact that these oscillations of brain activity correlate with the state of awareness and that their frequency and amplitude display reproducible features that are different in health and disease. In healthy conditions, oscillations of different frequency bands tend to minimize interference. This is similar to radio stations avoiding overlap between frequency bands to ensure clear transmission. During epileptic seizures, mutual entrainment, analogous to interference of radio signals, has also been reported. Moreover, in schizophrenia and autism, there is a loss of higher frequency oscillations. Most studies thus far have focused on the mechanisms generating the oscillations, as well as on their functional relevance. In contrast, our study focuses on what brain rhythms tell us about the functional network organization of the brain. Using a reverse-engineering approach, we construct abstract networks (virtual brains) that display oscillations of actual brains. These networks reveal that brain rhythms reflect different levels of hierarchical organization in health and disease. We predict specific alterations in brain connectivity in the aforementioned diseases and explain how clinicians and experimentalists can test our predictions.

## Introduction

Recent studies of electroencephalography (EEG) and magnetoencephalography (MEG) as well as of extracellular recordings (local field potentials) in acute brain slices have demonstrated that both macroscopic and microscopic neural networks exhibit multiple activity rhythms [1]–[5]. In the power spectrum, these rhythms appear as a number of frequency bands which are evenly spaced on a logarithmic scale, thereby reducing the potential for cross-talk (phase-locking) or mutual entrainment between frequency bands. Furthermore, the baseline of the power spectrum *P* decreases proportionally to the inverse of a power *α* of the frequency, 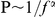, which is a feature common to many complex systems [1]. Using mathematical tools recently developed for solving inverse problems [6], as well as stochastic theory, we set out to reverse-engineer network configurations, or “virtual brains”, that recreate multi-oscillatory brain dynamics on a 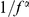
background. The virtual brains are not meant to model specific anatomical pathways or synaptic connections. Instead, they model functional coupling between elements (nodes) of an abstract network. We hypothesized, however, that the virtual brains would share common features, such as *functional* (not necessarily anatomical or synaptic) connectivity and dynamics, among themselves and also with actual brains. Characterizing these commonalities would in turn help us identify parameters and physiological features of healthy brains, such as the balance between functional excitation and inhibition, the relative number of highly connected nodes (hubs), the probability of finding certain structural motifs, etc. In addition, since the multi-oscillatory activity of the brain is known to be altered in disease, we also set out to reconstruct virtual brains that reproduce altered EEG and MEG patterns such as those observed in epilepsy [5], [7] and schizophrenia [8], [9]. We expected to find alterations of connectivity and dynamics in these altered virtual brains that would give us some insight into structural and physiological changes associated with the above pathologies.

## Results

The mathematical and computational details of our approach are explained in *Methods*. Briefly, following the approach of several authors [10]–[13], macroscopic brain activity was modeled as a linear multivariate stochastic system, which in its continuous version in time is equivalent to an Ornstein-Uhlenbeck process [14]

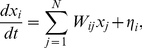
(1)where 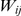 is the functional connectivity matrix, i.e. the coupling between the *j*-th and the *i*-th nodes; 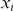 is the neural activity of the *i*-th node with respect to baseline, measured as the signal from the *i*-th EEG or MEG channel; 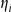 are the residuals (background, uncorrelated white noise) of the *i*-th channel; and *N* is the number of nodes (channels). The sign of *W_ij_* can be thought of as *functional* excitation and inhibition, although these do not necessarily represent excitatory and inhibitory synaptic connections at the cellular level. From a physiological perspective *W_ij_* can be thought of as the net effect of many excitatory and inhibitory synapses plus other neuromodulators converging onto the area associated with a node. The units of *W_ij_* are reciprocal of time, i.e. frequency units.

**Figure 1:**
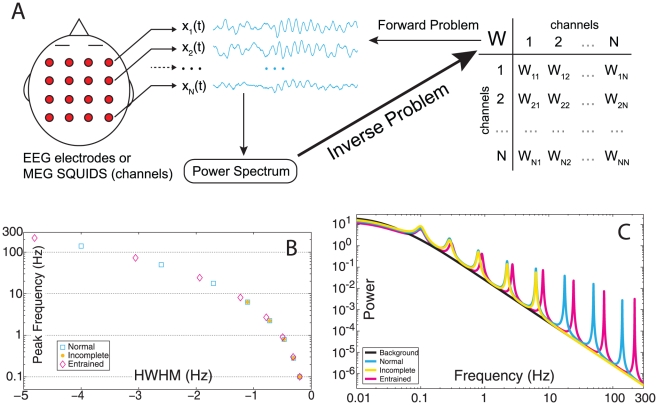
Reverse-engineering Virtual Brains. (**A**) Schematic representation of EEG/MEG recordings and the forward and inverse problems. *W* is the matrix of functional brain connectivity; *x*_i_(*t*) is the recording from the *i*-th channel. (**B**) Eigenvalues defining the peak frequency and half width at half maximum in the power spectrum. (**C**) Power spectrum.

If *W* is known, system (1) can be integrated numerically to simulate EEG or MEG recordings; this is the forward problem of calculating the dynamics from the connectivity matrix (Fig. 1A). Moreover, the power spectrum (PS, *P*(*ω*)) can be analytically calculated from (1), (see *Methods*). The solution to the inverse problem is more difficult: What is the underlying architecture, *W*, which gives rise to a particular power spectrum, *P*(ω)? This problem does not have a unique solution; in fact, there may be an infinite number of connectivity matrices (or “virtual brains”) which give rise to the same power spectrum. In spite of this, the inverse problem can still be rigorously investigated (see *Methods*). We note that the peaks of the power spectrum, *P*(*ω*), are determined by the eigenvalues of the connectivity matrix. In particular, the real part of the complex eigenvalues describes the half width at half maximum (HWHM) and the imaginary part describes the peak frequency. Thus, the eigenvalues of *W* can reciprocally be inferred from the power spectrum. This reduces our network reconstruction problem to an inverse eigenvalue-problem (IEP), which consists in finding a matrix with a given set of eigenvalues [6]. Following Chu and Golub [6], the IEP is formulated as a minimization problem in a matrix space. For different realizations of the initial condition, the algorithm converges to different matrices but all have the same prescribed spectrum. The reconstructed matrices contain no information about the spatial arrangement of the nodes; they only convey information about the network's graph structure (network topology). Adjacent elements in *W* do not represent connections between, for example, adjacent EEG electrodes.

Our algorithm is described in *Methods* and the complete Matlab code to obtain connectivity matrices from the power spectrum (Virtual Brain Generator) is available as supporting material and from our lab's webpage [15]. We have implemented this algorithm such that reconstructed networks generate brain dynamics in five different scenarios: 1) A power spectrum with evenly spaced frequency bands on a logarithmic scale (“Normal” group), a signature of long recordings of ongoing activity from healthy individuals [2]. 2) A spectrum with frequency bands that are rational multiples of each other leading to phase-locking (“Entrained” group). This form of “cross-talk” between frequency bands has been recently observed in magnetoencephalograms (MEG) of epileptic patients during seizures [5], [7]. Interactions between frequency bands can also occur in normal individuals but are transient and modulated in different behavioral tasks [16]. We note that MEG data and EEG data are complementary, since they measure different components of the same electromagnetic field generated by neural activity. 3) A spectrum lacking high-frequency peaks, as observed in patients with cognitive disorders, such as schizophrenia or autism [9] (“Incomplete” group). 4) As a first control, we also considered a 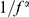 spectrum lacking any rhythmic oscillations, establishing an EEG baseline conserved across all groups (“Background” group). 5) As a second control for several analyses, we also considered EEG spectra where the frequency peaks had been randomly positioned with uniform probability on the logarithmic frequency axis (“Random” group). Each trial's spectrum in this group is different, as their peaks are shuffled independently. For each of these five scenarios, we constructed several (n = 20) virtual brains with 80 nodes each, so that we were able to analyze similarities and differences corresponding to normal and pathological conditions.

In order for (1) to have stable dynamics, i.e. to prevent that the traces of neural activity grow unbounded, the real part of every eigenvalue of *W* must be negative. Additionally, since the connectivity matrix is real, complex eigenvalues must exist as conjugate pairs, each of which prescribes a peak in the PS. The complex eigenvalues with positive imaginary part are shown in Fig. 1B for the Normal, Incomplete and Entrained groups. The remaining eigenvalues (not shown) are real valued and were randomly chosen from a uniform distribution bounded by the minimum and maximum real values of the Normal distribution (abscissa, Fig. 1B). The analytic power spectra resulting from the eigenvalues of *W* are displayed in Fig. 1C. These spectra recreate the defining features of real data from the studies cited above.

**Figure 2:**
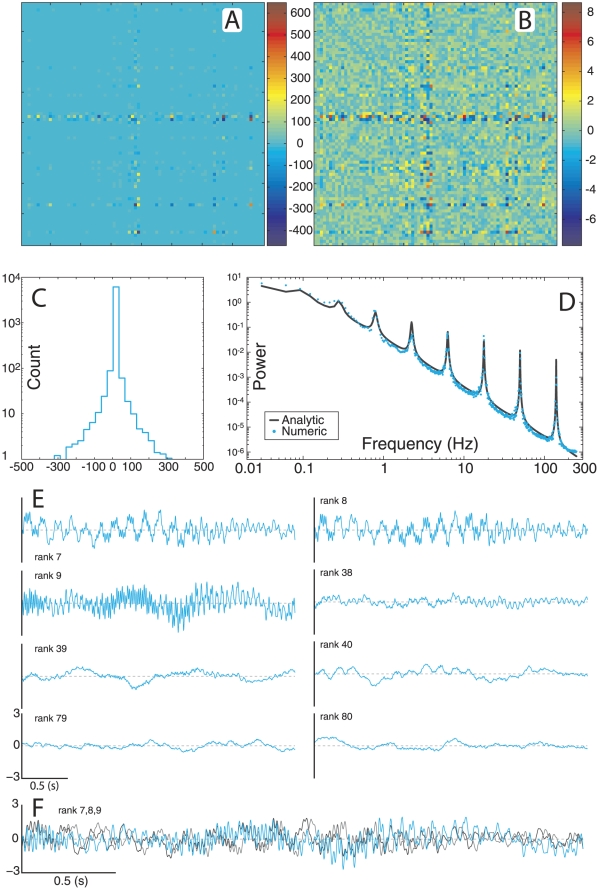
Characterization of the Normal Virtual Brains. (**A**) Example connectivity matrix from Normal group (**B**) and rescaled by cubic root. (**C**) Trial-averaged histogram of connection strengths. (**D**) Analytic and numeric power spectrum. (**E**) Selected epochs of dynamics from three regimes of TNS: low, medium and high ranks. (**F**) Magnified view of low-rank dynamics.

Having determined the eigenvalues of *W*, we ran the IEP to obtain *W* itself. In the case of the Normal group, connectivity matrices demonstrated sparse connectivity and a high degree of stratification of connective strengths (Fig. 2A). This stratification is still evident when rescaled by the cubic root (Fig. 2B) to enhance the contrast between weak and strong connections. Self connections (the diagonal) are strictly inhibitory (negative). We also note an overall balance between excitatory and inhibitory connections, observable in the trial-averaged distribution of connection strengths (Fig. 2C). The distribution of weights is long-tailed in both the positive and negative directions, and the vast majority of connections have minimal strength, especially when compared to the maximal connection strengths. The units of *W_ij_* are Hz, so the range of *W_ij_* values scales with the effective bandwidth of the prescribed power spectrum. For instance, the spectrum for the Normal group contains significant power between 0 and 300 Hz. Thus, the absolute value of most elements in *W_ij_* roughly spans the same interval, as shown in Fig. 2C.

Once the inverse problem has been solved, the forward problem can easily be computed by integrating (1) numerically. We compared the PS for the Normal group obtained analytically from expression (2) in *Methods* with the PS calculated numerically from the simulated channel traces (Fig. 2D). We observed good agreement between the analytic and numeric values. We also calculated a rank parameter based on the Total Nodal Strength (TNS), the sum of the absolute value of all the input and output connection strengths associated with a node. In this way, each node's TNS is calculated, and these values were ranked in descending order to yield TNS rank: *low rank nodes have the greatest TNS, high rank nodes the least*. We selected a 3-second epoch for channels selected from three TNS rank regimes: low (rank  = 7, 8, 9), medium (rank  = 38, 39, 40), and high (rank  = 79, 80). These dynamics (Fig. 2E) demonstrate common features to all virtual brains in the Normal group and change considerably between low and high ranked nodes. We observe network events, in which changes in dynamics correlate across channels, but we do not observe global entrainment; the dynamics of each channel are not strictly entrained to all other channels at all times (Fig. 2F). We also observe the appearance of spindle waves, wherein a transient oscillatory frequency, usually an increase from time-averaged dynamics, waxes, sustains, and wanes; observed most easily in the low rank nodes (Fig. 2E and 2F) corresponding to larger TNS.

**Figure 3:**
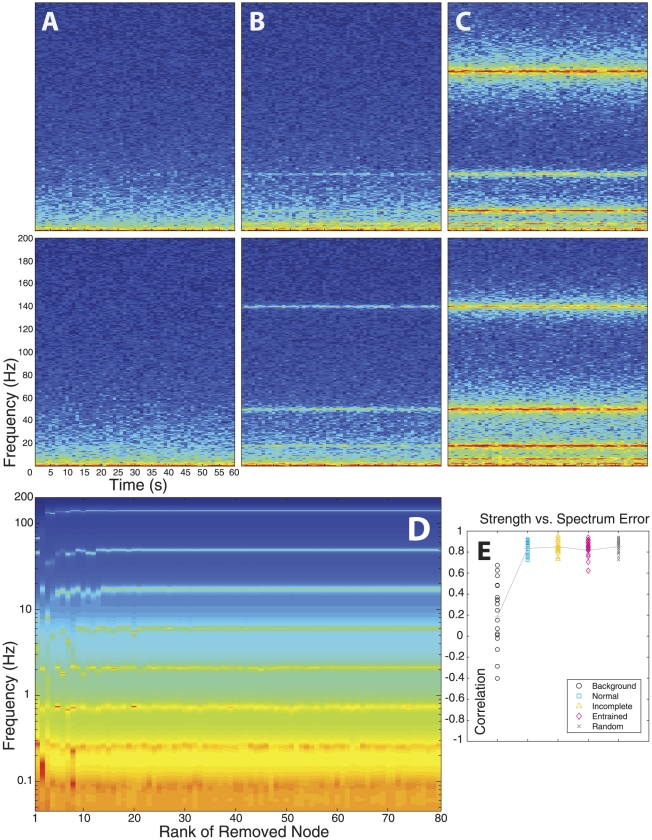
Nodal contributions to brain rhythms. (**A**) Spectrograms from high-ranked nodes, (**B**) from medium-ranked nodes, and (**C**) low-ranked nodes. (**D**) Power spectrum after the removal of an individual node of given rank. (**E**) Correlation of TNS rank to error between the unperturbed and the perturbed spectra from (**D**).

We next investigated the distribution of spectral power between channels. Spectrograms (time-resolved power spectra) were computed for the dynamics of nodes with different TNS ranks. We selected nodes from TNS rank regimes of high (rank  = 77, 79; Fig. 3A), medium (rank  = 39, 42; Fig. 3B), and low (rank  = 1, 3; Fig. 3C). We note that low TNS (high TNS rank) correlates with reduced high-frequency content (Fig. 3A), and note that such nodes show primarily slow frequency fluctuations, which are characteristic of pink noise. Nodes of medium TNS demonstrate the appearance of some medium and high frequency content (Fig. 3B), but the frequency characteristics are variable across nodes of similar rank. The spectrograms for low-ranked nodes (Fig. 3C) show the presence of all prescribed frequency content, with differing balance. Supporting Fig. S1 plots the analytical power spectra for various nodes from a matrix of the Normal group and each of the other groups studied below. We note again clear spectral differences for different nodes and a richer spectral structure for nodes with low TNS rank.

Given that spectral content of channel dynamics is distributed heterogeneously across TNS rank, we were interested in the role played by each node in affecting the dynamics observed in the whole network. To investigate this, we removed an individual node from the network, and analytically calculate the resultant perturbed PS. This is plotted in Fig. 3D, where the abscissa is the TNS rank of the singular removed node, the ordinate is the frequency and the color represents power from zero (dark blue) to highest (red). We see that removing low rank nodes affects the power spectrum the most, but no node's removal eliminates or adds peaks; rather, the removal of a node shifts and scales the existing peaks. The correlation between the rank of the removed node and the resulting spectral error was then calculated (Fig. 3E) as the normalized sum of absolute difference between the log_10_ of the spectra. For all cases where spectral peaks are prescribed, the correlation is high, signifying that nodes of increasing TNS rank contribute decreasingly to the PS. This suggests that the presence of brain rhythms is by itself indicative of a hierarchical network structure, where the removal of highly connected nodes (hubs) can have a major impact on the network dynamics. Consistent with this, in the Background group case, which lacks spectral peaks and hence brain rhythms, the correlation is significantly reduced, indicating a fundamental difference in stratification of nodes and channel dynamics.

**Figure 4:**
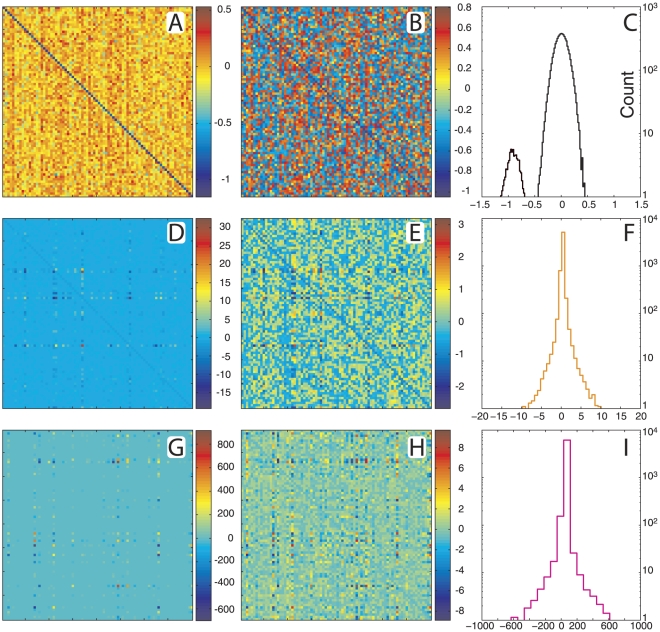
Characterization of non-Normal Virtual Brains. (**A**) Example connectivity matrix from Background group, (**B**) rescaled by cubic root (**C**). Histogram of connection strengths from Background group. (**D–F**) Similarly for the Incomplete group and for the Entrained group (**G-I**).

In addition to the Normal group, we also studied the remaining scenarios mentioned above. We first investigated the connectivity matrices of the virtual brains returned for these cases (Fig. 4). The Background group (Fig. 4A–C) differs the most from all other groups. The side lobe in the distribution of connection strengths is centered about −1 and accounts for the matrix diagonals (self connections), while the off-diagonal elements are centered about 0 (Fig. 4C). In the case of the Incomplete and Entrained groups (Fig. 4D–I), we note less dominance of the diagonal and greater connection strength stratification than in the Background case, but a more dominant diagonal and less stratification than in the Normal case. In all groups, the range of strength values scales with the effective bandwidth of the prescribed spectrum.

**Figure 5:**
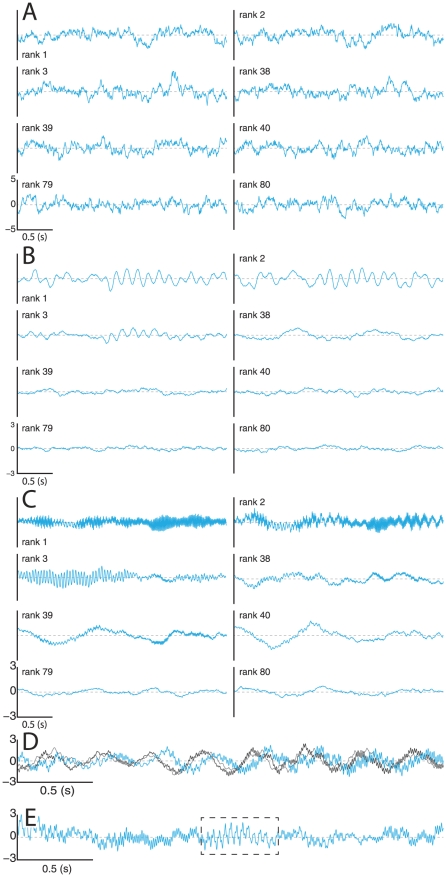
Dynamics for non-Normal Virtual Brains. (**A**) Selected epochs of dynamics for Background group from three regimes of TNS rank: low, medium and high rank. (**B**) Same for Incomplete group (**C**) and for Entrained group. (**D**) Superimposed traces from Entrained group. (**E**) Trace from Entrained group showing sharp waves.

We next investigated the dynamics evolved from these networks, in the same manner as with the Normal group. The dynamics for the Background group shows clear differences from any other group: dynamical features are the same regardless of TNS rank (Fig. 5A) and comprised solely of pink noise, where no oscillatory frequencies are preferred. The dynamics of the Incomplete group does show a dependence on TNS rank, where higher frequency content diminishes with increasing rank (Fig. 5B). However, the Incomplete group does not display the kind of transient activity (spindles) observed in the Normal group. We also observe weaker and less frequent network events. The Entrained group demonstrates the second greatest deviation from the behavior observed in the Normal and Incomplete groups. The Entrained dynamics again show a dependence on TNS rank (Fig. 5C), and additionally demonstrate overall channel entrainment (Fig. 5D), observable at 2.7 and 8.1 Hz, but existent across all frequencies. The Entrained group dynamics also demonstrate fluctuations reminiscent of “sharp waves”, which resemble an ictus in recordings from epileptic brains, observable in the dashed square shown in Fig. 5E.

The interactions between channel pairs can also be investigated via two spectral properties: the coherence and the phase spectrum (see §3 in *Methods*). For each frequency component, the former quantifies the fractional part of the signal power that is produced by the coupling with the other channel. The latter represents the relative phase lag of the oscillations in both channels at each frequency. Supporting Fig. S2 and S3 respectively show examples of coherence and phase lag for various pairs of nodes with different TNS rank from one virtual brain of each group. The spectra of those nodes are shown in Fig. S1. For all groups except the Background group, the coherence is high at each frequency, when both nodes in the pair have a low or moderate TNS rank. The coherence at high frequencies drops off when both nodes have high TNS rank. The drop-off is more pronounced for the Incomplete group. For the Background group, the coherence tends to decay quickly with the frequency. The phase spectrum (Fig. S3) shows that for most node pairs in all groups except the Background group, there is a fair degree of variability in the phase-lag as a function of the frequency regardless of the nodes' rank (note the wrap-around boundaries of the y-axis). Perhaps the most obvious feature is the tendency of pairs from the Background group to oscillate at high frequencies with a relative lag of a quarter of a cycle 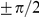.

**Figure 6:**
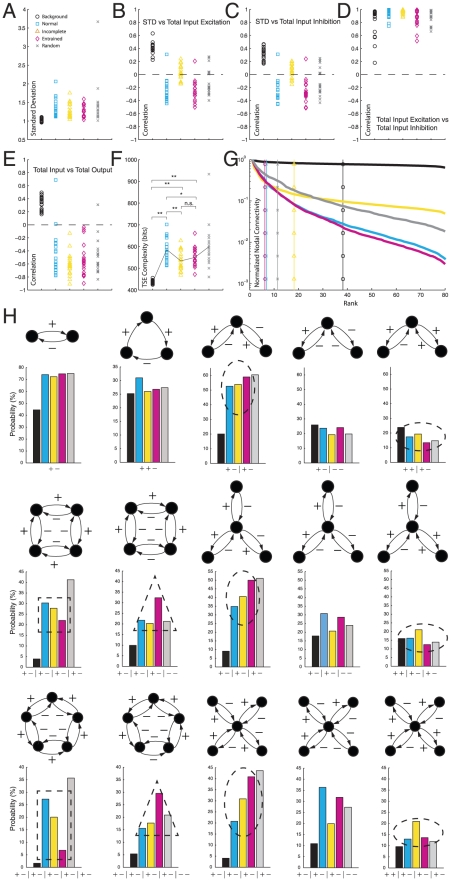
Comparisons between groups. (**A**) Channel-averaged standard deviations of dynamics for all virtual brains in all groups. (**B**) Nodewise correlation between standard deviation and total excitatory input. (**C**) Same for total inhibitory input. (**D**) Correlation between total input excitation and inhibition, and (**E**) between total input and total output. (**F**) TSE complexity. (**G**) Hierarchical structure of the virtual brains. The decay of TNS with TNS rank indicates a pronounced nodal hierarchy for the Normal and Entrained groups, that is less pronounced for the Incomplete group and absent in the Background group. (**H**) Relevant motifs appear with different probabilities in different groups.

We then investigated structural measures for the virtual brains in the five groups. First, we studied the standard deviation (mean amplitude) of channel fluctuations and its relationship with the inputs to the corresponding nodes. There was some variability with respect to the standard deviation averaged across channels within and between groups (Fig. 6A). We then computed for each virtual brain, the correlation coefficient between the standard deviation of each node's dynamics to two measures of the node's connectivity: the total excitatory input (Fig. 6B), and total inhibitory input (Fig. 6C). The totals were calculated by summing the absolute values of all excitatory or inhibitory input connections to a node. We found that in the Background group, there was a positive correlation between the overall amplitude of the signal fluctuations and the total amount of excitation received by the nodes (Fig. 6B). In contrast, there was a negative correlation in the Normal and Entrained groups. This is an important result for clinical analysis of ongoing EEG and MEG signals: nodes with smaller fluctuations tend to receive more excitatory drive. The virtual brains of the Incomplete case, however, show no correlation between fluctuation amplitude and total excitatory input. The Random group, investigated as a control, shows a spread across both positive and negative correlation. This is not surprising, since all virtual brains of this group have different spectra with randomly shuffled peaks.

Group-wise correlation between fluctuation amplitude and total inhibitory input match those of excitatory input (Fig. 6C), as the virtual brains from all groups show a strong correlation between the total excitatory input and the total inhibitory input impinging on a node (Fig. 6D). Moreover, there is a strong negative correlation between the net input to a node (sum of all elements in a row of *W*) and the net output (sum of all elements in the corresponding column of *W*) from that node (Fig. 6E). Thus, if the total input to a node is excitatory, the total output from that node tends to be inhibitory, and vice versa. The only exception is again the Background group. In brief, the properties shown in Figs. 6D and 6E ensure an overall balance between excitation and inhibition in the virtual brains, and hence their dynamical stability.

Next, we employed a measure developed by Tononi, Sporns, and Edelman (TSE) to quantify neural complexity based on the covariance matrices of the signals [17], which we analytically derived from each connectivity matrix (Fig. 6F; see also §5 in *Methods*). The Background group clearly shows smallest complexity. This implies that the presence of brain rhythms is associated with higher structural complexity. Among the other groups, except the Random group which is not physiologically relevant, we note that complexity is largest for the Normal virtual brains as compared to the Entrained and Incomplete virtual brains, which model pathological conditions. Remarkably, this suggests that neural complexity is also an indicator of brain fitness. The Normal group does not maximize TSE complexity, however, as at least one realization of the Random group displays larger complexity.

Early work on TSE complexity demonstrated that this measure is largest for hierarchical networks [17]. We next investigated the hierarchical structure of the virtual brains. We first plotted the TNS vs. the rank (Fig. 6G). The TNS was normalized, so that the TNS for the lowest rank was one, and averaged across all virtual brains from the same group. Vertical lines in Fig. 6G indicate the 50^th^ percentile of ranked TNS, i.e., the nodes up to that rank account for 50% of the total nodal strength in the whole network. Hierarchical networks are characterized by a steep decay of TNS with the rank and by a low 50^th^ percentile of ranked TNS, and we observe such a decay for the Normal and Entrained groups. The virtual brains from the Incomplete group display only a moderate hierarchical structure, whereas the Background group shows no hierarchical structure whatsoever. This suggests that brain rhythms reflect a hierarchical network where a few nodes are strongly connected to many, whereas most nodes are only weakly connected to the rest.

Along the lines of previous studies [18], we then asked what specific structural motifs (network graphs and subgraphs) may account for the differences in TSE complexity we observed. We focused on connectivity patterns among nodes below the 50^th^ percentile of ranked TNS, i.e. among the nodes with greater connective strength. Even in this case, the number of subgraphs to consider is phenomenally high. However, due to the nature of our problem, some educated guesses on relevant motifs can be made. For instance, from linear algebra we know that at least two nodes are necessary in order for system (1) to have a spectral peak, which is the minimum number of nodes required to have a complex eigenvalue of *W* and its conjugate. For the same reason, in order to study interactions between two spectral peaks, the minimum number of nodes to consider in a subgraph is four. From linear algebra we also know that a simple way of obtaining complex eigenvalues consists in having graphs with a circulant connectivity matrix (a particular case of a Toeplitz matrix), which represents a cyclic loop. Taking all these pieces of information into account, we considered subgraphs with up to five nodes representing cyclic loops with both, single and reciprocal (feedforward and feedback) connections. In addition, we considered subgraphs with radial connectivity, in which a given node is linked to other nodes with reciprocal connections, but the connections among these nodes are not considered. All these subgraphs and their probabilities of appearing in all scenarios are shown in Supporting Figs. S4 and S5. An explanation of how the probabilities were calculated is presented in *Methods*. A selection of relevant network motifs is shown in Fig. 6H. We first note that for all groups with spectral peaks, the most common motif of two reciprocally coupled nodes is the one having forward excitation and feedback inhibition or vice versa. This helps maintain the overall balance between excitation and inhibition. Second, we note that a given type of cyclic loop with reciprocal connections is more frequent in the Normal group as compared to the Incomplete and especially the Entrained group. This is the cycle in which each feedforward excitatory connection has inhibitory feedback (Fig. 6H, dashed squares). As the number of nodes in the cycle increases, the predominance of this subgraph increases in the Normal group as compared to the Entrained and Incomplete groups. Third, we note that for the Entrained group, cyclic subgraphs in which two nodes are connected through reciprocal inhibition and the rest are counterbalanced have a much higher probability than in the Normal and Incomplete groups (Fig. 6H, dashed triangles). Fourth, we note that radial subgraphs in which each feedforward excitatory connection is counterbalanced with feedback inhibition are less probable in the Normal group, as compared to the Incomplete and even more clearly to the Entrained group (Fig. 6H, dashed prolate ovals). Fifth, in the Entrained group, radial motifs in which two nodes are connected through reciprocal excitation, but the remaining connections are counterbalanced, are much more probable than in the Normal and Incomplete groups (Fig. 6H, dashed oblate ovals). In brief, the three groups that are physiologically relevant possess clearly different probabilities of certain motifs. In particular, the Normal group is characterized by cyclic graphs with balanced feedforward excitation and feedback inhibition, as well as by radial motifs in which the symmetry between excitation and inhibition is broken. Deviations from these structural patterns may be indicative of a pathological condition, as suggested by the results from the Incomplete and Entrained groups.

## Discussion

The existence of brain rhythms has intrigued neuroscientists since the early observations of electrical brain activity by Richard Caton in the late 19^th^ century [19], and later by Hans Berger, who recorded and documented electrical oscillations in the human brain with an unprecedented level of detail in the beginning of the 20^th^ century, despite the technological constraints of the time [19], [20]. The functional relevance of network oscillations has been debated ever since. Substantial progress has been made over the last few decades with experiments and computational models in our understanding of the biophysical mechanisms generating brain rhythms, and in particular, on neuronal synchronization [1], [3], [4], [21]–[24]. However, the functional role of those oscillations is still unclear. The approach taken in this paper was to investigate functional aspects of oscillatory brain dynamics, rather than the mechanisms underlying those oscillations. Our focus was not on *how* oscillations are generated or *why*. Instead, we started off with an agnostic approach and focused on *what* oscillations tell us about functional brain connectivity.

To this end, we applied a reverse-engineering strategy using a generic mathematical model of high-dimensional stochastic dynamics, namely, a multivariate Ornstein-Uhlenbeck process (1). It is important to mention that there is no contradiction between such a stochastic linear model and the fact that brain dynamics are strongly nonlinear, because we do not intend to model neuronal dynamics per se. Instead, we use a phenomenological model of the recorded signals, which are electromagnetic fields that do superimpose linearly. An analogue dichotomy takes place in weather forecasting. Although the dynamics of air masses is turbulent, chaotic and therefore, unpredictable, when considered over a large area the flow of air masses becomes predictable within a time window of a few days. This coarse dynamics of air masses fits well a linear multivariate stochastic process, which can then be used to accurately forecast variations and co-variations of air pressure and temperature in several locations [25]. In neural models, the strong nonlinear dynamics of single neurons when averaged over a fairly large spatial range displays regularities that make a stochastic linear model suitable for the description of large-scale activity. We also note that nonlinear neuronal networks, like those based on the celebrated Wilson-Cowan model [26], [27] and the neural-mass models [28]–[34] frequently possess hyperbolic equilibria. Linearization of the dynamics around a hyperbolic equilibrium leads to model (1) when stochastic perturbations are included. A recent paper has taken advantage of this fact to investigate the link between connectivity and spontaneous activity patterns in a neural network model [13].

Using our reverse-engineering approach, we have demonstrated that the power spectrum of neural recordings conveys significant information about the functional, though not necessarily anatomical, connectivity of the brain. We have first reconstructed virtual brains displaying multi-oscillatory dynamics over a 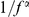
background that resemble those of field potentials from EEG traces and magnetic fields from MEG recordings. We have then shown that the presence of these brain rhythms is indicative of a hierarchical network structure with high complexity, in which certain motifs with reciprocal connections are more probable than others. We have finally shown that alterations of the multi-oscillatory activity lead to a reduction of neural complexity, changes in the hierarchical structure, and changes in the probability of finding certain motifs. Alterations were imposed either by reducing the high-frequency content or by allowing cross-talk (in the form of entrainment) between different rhythms. Neural complexity, as defined by Tononi, Sporns and Edelman [11], can thus be regarded as a measure of brain fitness. This is consistent with recent MEG studies revealing decreased functional connectivity, measured as large-scale coordinated dynamics (long-range phase synchronization) in autism-spectrum disorders [35].

Our results complement previous models of neural dynamics underlying field potential recordings. Recent computational work by Miller et al. [36] demonstrates that neuronal inputs with Poisson statistics plus synaptic filtering and passive Ohmic filtering in the tissue can account for the power-law drop-off of the spectral background observed in subdural recordings. Without Ohmic filtering, the drop-off decreases as 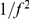, but with Ohmic filtering, which in their model acts as a low-pass filter, the drop-off decreases as 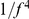. An important consequence is that the drop-off does not reflect any specific features of the connectivity but rather is a general property of random baseline activity. Our results are consistent with that model in the sense that the spectral background does not convey any information about structural complexity of the underlying networks. Indeed, virtual brains obtained from the Background group are not hierarchically organized. On another note, the drop-off of the spectral background decreases as 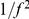 in our model, which is a consequence of system (1) being an Ornstein-Uhlenbeck process when driven with white noise. Indeed, Ornstein-Uhlenbeck processes are general models of Brownian motion and hence, display a 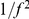
drop-off. However, if system (1) is driven with low-pass filtered noise (e.g. 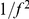
noise), then the spectral drop-off decays as 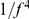, as shown in Supporting Fig. S6. We also show in Fig. S6 that in the low frequency range, the slope of the spectrum on a logarithmic scale can be made steeper or shallower by increasing the density of real eigenvalues to the right, i.e. less negative (Fig. S6B) or to the left, i.e. more negative (Fig. S6C), respectively (dashed lines indicate the median of the eigenvalue distribution).

When driven with white noise, the statistics of model (1) are Markovian and Gaussian [14]. Transient spindle-like oscillations represent chance excursions in a Gaussian field that fade out with an exponential decay. The fact that the model displays these realistic activity patterns is in our opinion a good validation of our model despite its simplicity, and suggests that at least some spindle-like network events may indeed have a probabilistic nature in real brains. There is, however, evidence for non-Gaussian statistics in EEG recordings. Recent computational models [37], [38] based on experimental observations [39] show that nonlinear thalamocortical coupling may underlie the bistability observed in alpha rhythms, a phenomenon that cannot have Gaussian statistics, and hence, cannot be accounted for by our model. We note, however, that our model does not take into account thalamocortical coupling (or any other anatomical structures) explicitly, which may certainly enrich brain dynamics with strongly non-linear and non-Gaussian features.

Regarding the size of the networks investigated, we chose 80 nodes as a trade-off between computing time and network size, since the time needed to solve the inverse problem increased exponentially with network size. We felt 80 nodes was an appropriate network size as it is much larger than the number of electrodes in EEG studies (typically 20). Current MEG studies may use up to 300 SQUIDS but the signals recorded in neighboring sensors overlap considerably, and thus fewer distinct sources can be resolved after independent component analysis. In our simulations, a hierarchical structure was already apparent in virtual brains of 20 nodes. To test whether this was an artifact of small network size, we progressively increased the size to 40, 50, 60 and finally 80 nodes. Looking at motifs in the 80-node networks, we note a prevalence of bidirectional links. This is consistent with structural connectivity studies in the macaque showing that 85% of the cortical fiber tracts are bidirectional [40]. We also investigated solutions with size 85, 90, and 100 nodes, and found that the convergence of the inverse problem required a prohibitive amount of computational time, and networks did not display any marked changes from size 80 networks with the measures investigated.

Our model provides some insight into the functional role of oscillations. We found, for instance, that the virtual brains do not have pace-making nodes. The oscillations are distributed across nodes but not all nodes oscillate with the same frequencies and the nodes that are strongly connected tend to display more frequencies and richer patterns, as shown in Fig. 5 and Supporting Fig. S1. In a cognitive context, this inhomogeneous distribution of brain rhythms across nodes supports the idea that oscillations may act as a mechanism for linking perceptual information across sensory and associative areas, as stated by the so-called binding hypothesis [41].

The relationship between our model and cognitive function is also apparent from another perspective. Current views on the brain-mind continuum propose that in certain neuropathologies there is increased excitability but decreased variability (activity fluctuations), which indicates an alteration of functional coupling compared to the healthy brain [42]. This is particularly clear during epileptic seizures, when the mean excitability increases but the activity becomes less variable in time and space. More generally, the inverse relation between excitability and variability (more of one, less of the other or vice versa) may be a fairly important property of the brain, which is captured by the Normal and Entrained groups of virtual brains. For these two groups, the standard deviation of field-potential fluctuations correlates inversely with the excitatory drive to a node (Fig. 6B). Interestingly enough, this correlation does not exist in the Incomplete group, which models neuropathologies associated with cognitive deficits.

We find it quite remarkable, that the channel-averaged power spectrum alone contains sufficient information to reconstruct virtual brains which are clearly distinct across groups. This suggests that the spectral features shown in Fig. 1D, namely evenly spaced peaks on a logarithmic frequency band, are quite fundamental and carry significant information about the brain's function and structure, as previously speculated by other authors [1]–[3]. One expects, however, that if instead of using the channel-averaged power spectrum one used the spectrum of each channel, as well as the cross-spectra, one may be able to reconstruct virtual brains with many more features. For that, however, one would need data sets from actual recordings, since those spectra are not found in the literature with sufficient detail. Another extension of the approach presented here would consist in endowing the connectivity matrix with spatial relationships. The relative position of the nodes (recording channels) is irrelevant to our study, and our current algorithm does not take it into account. In order to implement all these extensions, our reverse-engineering approach would need to be expanded, thereby increasing its already high computational cost.

We predict that some of our results on virtual brains will also be found in actual brains in health and disease. Clinical and experimental groups interested in testing our predictions may proceed in two steps. They will first obtain *W* by linear regression of the recorded data and their time derivative to system (1). They will then perform the analyses displayed in Fig. 6, in particular 6F–H, and compare their results with ours. Future work should also explore the link between functional network connectivity and anatomical connections between neuronal populations, which was not the scope of our project but is a natural extension of it. This challenging task, however, will require a multiscale modeling approach, based on accurate descriptions of the anatomy and physiology of the brain that include synaptic and dendritic filtering, as well as axonal delays, which are known to play an important role in resting brain fluctuations [43]. Previous work by other authors indicates how different levels of organization can be integrated successfully into models of neural activity [29]–[31], [33], [34], [44], [45], which can explain the emergence of spatiotemporal patterns of EEG activity. These approaches, in particular, neural-mass models [29], [33], [34], [44], [46], [47] are promising to find out the relationship between the functional connectivity matrix investigated here and the physiological parameters referred to above. We note that in order to investigate spectral properties of field potentials, like the frequency and dampening of network oscillations, neural-mass models are linearized around the steady state [44], [47]. In addition, fluctuations around the mean voltage are assumed to be driven by white noise [47]. In a spatially discrete rather than a continuum neural-mass model, this would lead to a linear stochastic equation equivalent to model (1). Proceeding this way, one may find a useful link between neural-mass models and ours, since the connectivity matrix will be determined by the parameters of the neural-mass model that account for synaptic and dendritic filtering as well as axonal delays and thalamocortical coupling. This connection between neural-mass models and our model deserves further attention in future studies.

## Methods

### 1. The forward problem: From network connectivity to spectral properties of network dynamics

Recordings of macroscopic brain activity can be modeled as a linear multivariate stochastic process [12], [17]. The modeled dynamics are multivariate because different channels (e.g., EEG electrodes or MEG “SQUID” detectors) capture different, although not totally independent, signals. The dynamics are stochastic because the signals can be forecast only over a short-time window and up to a given confidence level. And finally, they are linear because the electromagnetic fields and potentials generated by the activity of thousands of neurons under the scalp superimpose linearly. The phenomenological model of EEG, MEG or field-potential signals is given by equation (1). If the element *W_ij_* is positive, the rate of change of activity in *x_i_* increases when the activity of *x_j_* is above its mean, which is zero. If *W_ij_* is negative, the rate of change decreases. If *W_ij_* is zero, the activity of *x_i_* is not directly influenced by the activity in *x_j_*. Hence, the sign of *W_ij_* can be thought of as functional excitation and inhibition, although these do not necessarily represent excitatory and inhibitory synaptic connections on a cellular level. As a result, the network nodes are not subject to Dale's principle, so that a given node can excite and inhibit different nodes at the same time. Note also that the units of *W_ij_* are reciprocal of time, i.e. frequency units (Hz in our case). We emphasize that *W_ij_* are not synaptic connections but represent functional coupling between brain areas from which the neural signals are recorded.

From (1), the direct or forward problem is easily solved: *Given a connectivity matrix W, what is the power spectrum P(ω) averaged across EEG channels?* Using matrix notation, the answer is:
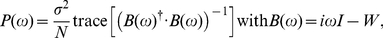
(2)where the dagger “†” denotes the conjugate transpose, *i* is the imaginary unit, *I* is the identity matrix, *N* is the number of channels (nodes) and σ is the standard deviation of the residuals. The solution to the inverse problem is much more difficult: *What is the underlying architecture, W, which gives rise to a given power spectrum, P(ω)?* This problem does not have a unique solution. In fact, there may be an infinite number of connectivity matrices (or “virtual brains”) leading to the same average power spectrum of the EEG. We hypothesized that these matrices would have important features in common.

### 2. The inverse problem: From spectral properties of network dynamics to network connectivity

We note that the peaks of the power spectrum (2) are determined by the eigenvalues of the connectivity matrix, *W*. Moreover, the eigenvalues can be inferred from the power spectrum, *P(ω)*. This is a consequence of (1) being a multivariate Ornstein-Uhlenbeck process, in which *W* is the drift operator. In effect, it is well-known in stochastic theory that the power spectrum of an Ornstein-Uhlenbeck process is a superposition of Lorentzians whose location on the frequency axis, width and height are determined by the eigenvalues of the drift operator [14]. In particular, the real part of the complex eigenvalues describes the half width at half maximum (HWHM) and the imaginary part describes the peak frequency in radians per time unit. Reciprocally, the eigenvalues of *W* can be inferred from the power spectrum, *P*(ω). This reduces our network reconstruction problem to an inverse eigenvalue problem [6], which consists in finding a matrix with a given set of eigenvalues. Because there might be an infinite number of matrices with a given eigenvalue set, the problem needs additional constraints, like prescribing some entries of the sought matrix. This can be done, for example, by randomly setting a fraction of entries to zero, i.e. by setting a level of *sparseness* for the network connectivity. The convergence of the algorithm is facilitated by prescribing a lower bound for the sparseness of the connectivity matrix (number of zero entries). This is a loose constraint, though, since the matrices that the algorithm converges to are much sparser than the lower bound prescribed (35%). To be more precise, the reconstructed matrices do not contain exactly zero entries but the values of a large fraction of entries are negligible compared to the values of the dominant nodes (Figs. 2A, 2B and 4). In that regard, the matrices are indeed sparse. Even with the constraint on sparseness, there may be a large number of matrices with the same eigenvalue set. It may also occur that there is no matrix with those prescribed entries and eigenvalues. Following Chu and Golub [6], a workaround for these issues is to define the solution in the least square sense: The matrix we are looking for, *W*, minimizes the distance to its projection, Π(*W*), onto the subspace of matrices with the prescribed entries. This distance in the matrix space represents a residual, or error function to be minimized, which defines a gradient flow in the space of matrices. Starting with a random initial condition, the flow converges in the least-square sense to a matrix with the prescribed eigenvalues and the prescribed entries. More technically, let *Λ* be a diagonal matrix containing the set of eigenvalues that determine the power spectrum. Then, define the matrix function *k*(*X*) as 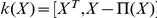
_,_where the square brackets denote the commutator, i.e. 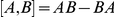. Finally, let *V* be a matrix of the same size as *W*, satisfying the following differential equation
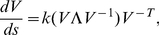
(3)starting with a random matrix, *V*(0) as initial condition. The numerical integration of equation (3) allows us to obtain a connectivity matrix (virtual brain). In effect, as 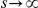, the product 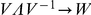
_._ To initialize the integration, *V*(0) was chosen as a random matrix, with values taken from a set which was defined to have a width of one and a random center value between −0.5 and 0.5; thus, initial values were ultimately bounded between −1 and 1, with each trial having its own particular bounds for the distribution of initial values in *V*(0), a distribution which was almost assuredly not symmetric about zero. Candidate random matrices (size 80×80) were created until *V*(0) had a condition number less than 200 (in order to assure numerical stability and reduce computation time), and then the integration was initialized. Integration was performed with the built-in Matlab ODE solver ode15s. The integration was halted when the residual given by
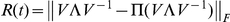
fell below a determined threshold of value 10 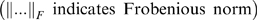. The residual is a measure of error between the requested form of the connectivity matrix and the form at each iterative step, where the iterative solution retains the prescribed eigenvalues exactly and approaches the prescribed connective strengths, and is thus proportional to the size of the connectivity matrix and the number of prescribed entries. At the beginning of the optimization problem, i.e. for random matrices of square dimension 80 and condition number less than 200, the residual is in the range of several orders of magnitude (∼10^4^). In the first few iterations the residual decreases very rapidly; however, the convergence during a given step decreases quickly with each subsequent iteration. Empirically, we observed that for networks of size 80, below a residual value of 10 the convergence rate is extremely slow, so further iterations do not improve the solution notably.

For different realizations of the initial condition, the algorithm converges to different matrices, but all have the prescribed eigenvalue spectrum. We noted that the method returns numerically identical connectivity matrices when fed with identical initial conditions. We also observed only small variation in the final form of connectivity matrices when initial conditions were minimally varied. Despite the high degree of variability in the initial conditions, the IEP converged to matrices whose entries where symmetrically distributed around zero and shared remarkable structural features, as shown in *Results*.

### 3. Analytical calculation of the coherence and phase spectrum

Two important spectral quantities, the coherence and the phase spectrum, can be analytically calculated from equation (1). In vector notation, equation (1) reads 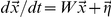. After taking its Fourier transform, equation (1) in the frequency domain becomes 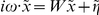, where *i* is the imaginary unit; 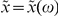 is the Fourier transform of 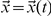 and 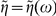 is the Fourier transform of 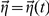. Thus, the signals in the frequency domain are given by
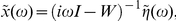
where *I* is the identity matrix. The cross-spectrum matrix is then given by
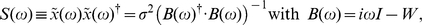
where we have used that for white noise 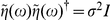
_,_ and *σ* being the standard deviation of the white noise process. Note that the power spectrum of each channel is given by the diagonal elements of this matrix and hence, averaging the diagonal we arrive at expression (2), the channel-averaged power spectrum. The *coherency* between the *i*-th and the *j*-th channel is defined as the normalized cross-spectrum 
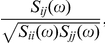
which yields a complex number for each value of the frequency. Finally, the *coherence* is defined as the magnitude squared of the coherency, and the phase (angle) of the coherency at each frequency defines the *phase spectrum* [48]. Examples of coherence and phase spectrum analyses are shown in Supporting Figs. S2 and S3, respectively.

### 4. Analysis of neural complexity

Neural complexity was calculated as proposed by Tononi, Sporns and Edelman [17], using the Matlab code provided by Sporns on one of his Websites [49] that computes neural complexity from the covariance matrix of the dynamics. The covariance matrix was calculated from (1) as reported below (see §5). Neural complexity was different across groups and the statistical significance of the differences was assessed using bootstrap analysis.

#### Bootstrap analysis for Fig. 6F

The significance tests were run in Matlab (version 2010a) using bootstrap analysis. We wanted to test whether the difference of the means of any two groups was significantly different. Parametric (e.g. t-tests) and non-parametric (e.g. Wilcoxon ranksum) tests were not applicable for our data sets, as the implicit assumptions of these tests were not satisfied. Bootstrap analysis was performed as follows. We first gathered the 20 data points from each group into one group with 40 data points. We then drew 20 points randomly to form a new group, allocating the remaining 20 data points to form a second group. This way we created random surrogate data sets from which the difference of the means was calculated. We then iterated this process one million times to build a probability distribution of the difference of the means for the surrogate data. If the difference between the means of the two groups with the actual data was larger than the 95 percentile of this distribution, then that value was considered to be statistically significant. The p-value was calculated as the integral of the distribution from the left end (−∞) up to the actual value. Significant values were indicated in the figures with one asterisk if 0.01≤p<0.05, and with two asterisks if p<0.01.

### 5. Covariance of nodal dynamics and functional connectivity

We now show how to calculate the covariance matrix of neural activity from the connectivity matrix. Starting with (1), the covariance matrix is defined as
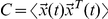
where the brackets indicate temporal average. From stochastic theory [14], we know that the relationship between the covariance matrix and the drift operator in (1) is given by
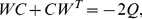
(4)where *Q* is the covariance matrix of the background noise, which in our case is uncorrelated across channels and with standard deviation σ, thus, we have 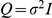. Let *D* be a diagonal matrix with the eigenvalues of *W*, so that *D_ii_* = *λ_i_*, and *L* the matrix of eigenvectors, so that the eigenvalue decomposition of *W* reads 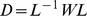
(5)


Define 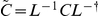
and 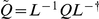, where the dagger “†” denotes the conjugate transpose. Then, upon substitution of (5) in (4) one obtains 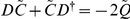, or equivalently, 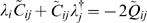, which implies that
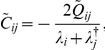
and finally
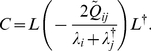
(6)


A similar derivation has been previously reported in [13]. In that paper the temporal evolution of (1) was discretized with a finite time step Δ*t*. The covariance matrix obtained there is identical with (6) in the limit of 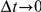.

The diagonal of the covariance matrix contains the variance of the fluctuations in each channel. Thus, the standard deviation or mean amplitude of the field-potential fluctuations is just the square root of the diagonal elements. Hence, equation (6) allows us to calculate amplitude fluctuations directly from the connectivity matrix without integrating (1). This method was used for the analyses displayed in Figs. 6A–C.

### 6. Searching for structural motifs and computing their probabilities

We focus on connection patterns between the most relevant nodes in the network, i.e. those below the 50^th^ percentile of *ranked* TNS. Our search for specific motifs was based on educated guesses as described in the text. Obviously, other relevant motifs may also exist that have not been considered. The motifs that were investigated are shown in Figs. 7 and 8. A selection of significant cases is also shown in Fig. 6H in the main manuscript. We note that only the sign of the connections is taken into consideration. In reciprocal connections, we do not distinguish between the direction of excitation and inhibition. For instance, node A exciting node B, which inhibits node A 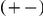, is equivalent to node A inhibiting node B, which excites node A 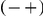. Moreover, motifs obtained by permutations of the reciprocal loops are counted as realizations of the same motif. For example, a motif with two reciprocal loops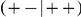 was considered the same motif as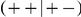. Taking into account these rules, we calculated the number *M* of all possible motifs with *n* nodes, *m* reciprocal loops (or *m* directed connections in the case of non-reciprocal connections), and a given topology (radial or circular) in the 20 virtual brains of each group. The probability, *P* of a given motif, is then the number of times, *F* that it appears relative to the total number of possible motifs,
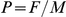
_,_ expressed as a percentage.

## Supporting Information

Figure S1 Power spectra for various nodes of different ranks. **(A)** Low rank (strongly connected) nodes. **(B)** Medium rank nodes. **(C)** High rank (weakly connected) nodes.
(EPS)

Figure S2 Coherence between pairs of nodes shown in Fig. S1. **(A)** Low rank node versus low, medium and high rank nodes. **(B)** Medium rank node versus low, medium and high rank nodes. **(C)** High rank node versus low, medium and high rank nodes.
(EPS)

Figure S3 Phase spectrum for the pairs of nodes shown in Fig. S1. **(A)** Low rank node versus low, medium and high rank nodes. **(B)** Medium rank node versus low, medium and high rank nodes. **(C)** High rank node versus low, medium and high rank nodes.
(EPS)

Figure S4 Relevant motifs with two, three and four nodes.
(EPS)

Figure S5 Relevant motifs with five nodes.
(EPS)

Figure S6 Eigenvalue distributions and background power spectrum for networks driven with white and low-pass filtered (colored) noise. The low-pass filter time constant was τ = 10 ms. **(A)** Uniform distribution of real eigenvalues. **(B)** Eigenvalue distribution skewed to low negative eigenvalues. **(C)** Eigenvalue distribution skewed to high negative values.
(EPS)
